# Diffusion Restriction Comparison between Gleason 4 Fused Glands and Cribriform Glands within Patient Using Whole-Mount Prostate Pathology as Ground Truth

**DOI:** 10.3390/tomography8020053

**Published:** 2022-03-02

**Authors:** Savannah R. Duenweg, Xi Fang, Samuel A. Bobholz, Allison K. Lowman, Michael Brehler, Fitzgerald Kyereme, Kenneth A. Iczkowski, Kenneth M. Jacobsohn, Anjishnu Banerjee, Peter S. LaViolette

**Affiliations:** 1 Department of Biophysics, Medical College of Wisconsin, 8701 Watertown Plank Rd., Milwaukee, WI 53226, USA; sduenweg@mcw.edu (S.R.D.); sbobholz@mcw.edu (S.A.B.); 2 Department of Biostatistics, Medical College of Wisconsin, 8701 Watertown Plank Rd., Milwaukee, WI 53226, USA; xfang@mcw.edu (X.F.); abanerjee@mcw.edu (A.B.); 3 Department of Radiology, Medical College of Wisconsin, 8701 Watertown Plank Rd., Milwaukee, WI 53226, USA; alowman@mcw.edu (A.K.L.); mbrehler@mcw.edu (M.B.); fkyereme@mcw.edu (F.K.); 4 Department of Pathology, Medical College of Wisconsin, 8701 Watertown Plank Rd., Milwaukee, WI 53226, USA; kaiczkowski@mcw.edu; 5 Department of Urology, Medical College of Wisconsin, 8701 Watertown Plank Rd., Milwaukee, WI 53226, USA; kjacobsohn@mcw.edu; 6 Department of Biomedical Engineering, Medical College of Wisconsin, 8701 Watertown Plank Rd., Milwaukee, WI 53226, USA

**Keywords:** MP-MRI, diffusion, cribriform glands, prostate cancer, Gleason

## Abstract

The presence and extent of cribriform patterned Gleason 4 (G4) glands are associated with poor prognosis following radical prostatectomy. This study used whole-mount prostate histology and multiparametric magnetic resonance imaging (MP-MRI) to evaluate diffusion differences in G4 gland morphology. Fourty-eight patients underwent MP-MRI prior to prostatectomy, of whom 22 patients had regions of both G4 cribriform glands and G4 fused glands (G4CG and G4FG, respectively). After surgery, the prostate was sliced using custom, patient-specific 3D-printed slicing jigs modeled according to the T2-weighted MR image, processed, and embedded in paraffin. Whole-mount hematoxylin and eosin-stained slides were annotated by our urologic pathologist and digitally contoured to differentiate the lumen, epithelium, and stroma. Digitized slides were co-registered to the T2-weighted MRI scan. Linear mixed models were fitted to the MP-MRI data to consider the different hierarchical structures at the patient and slide level. We found that Gleason 4 cribriform glands were more diffusion-restricted than fused glands.

## 1. Introduction

Prostate cancer (PCa) accounts for 26% of new cancer diagnoses in men, making it the most common non-cutaneous cancer in men. An estimated 249,000 new cases of PCa will be diagnosed in 2021, although not all cases have a high risk of metastatic potential [1]. Prostate cancer is graded using the Gleason grading scale, which assigns a score corresponding to the morphologic characteristics of the two most predominant glandular patterns. These patterns assign patients into five Grade Groups (GG) to predict prognosis [2]. Predominantly Gleason 3 (G3) cancers carry low metastatic risk and are now monitored through active surveillance. Clinically significant cancers, including Gleason grade 4 (G4) and grade 5 (G5), are more aggressive and are more likely to progress and cause death [3].

**Figure 1:**
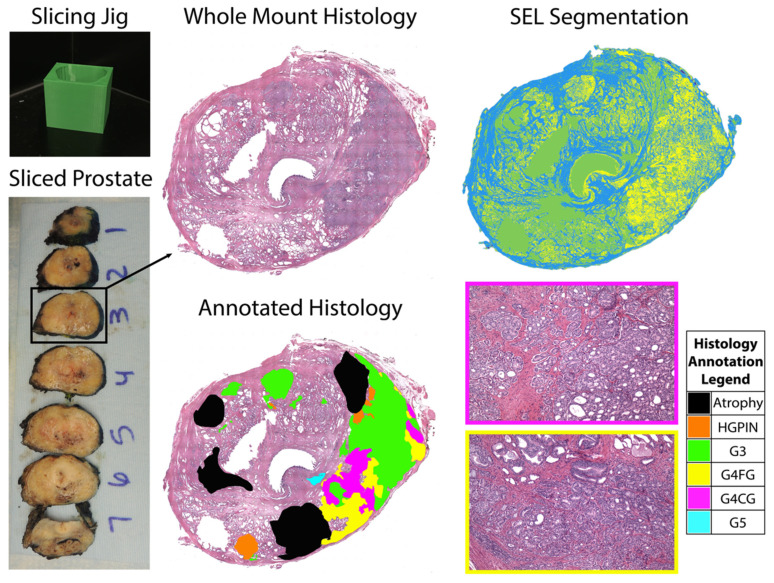
Patient-specific slicing jigs are modeled to match the slice thickness of the T2-weighted image (**left**). The slices are H&E stained, digitized, and annotated for different Gleason patterns (**middle**). Representative tiles from areas of Gleason 4 cribriform glands and fused glands (G4CG and G4FG, respectively) can be seen on the (**right**, **bottom**). Prostate tissue segmentation (SEL) was performed to create masks for the stroma (blue), epithelium (yellow), and lumen (green) (**top**, **right**).

Since the original 2011 discovery that biochemical recurrence was strongly related to the presence and extent of cribriform glands [4], the cribriform pattern has no longer been considered a type of Gleason 3 cancer and was entirely moved to Gleason 4. The 2014 International Society of Urological Pathology (ISUP) consensus conference included cribriform along with fused and poorly formed glands as defining Gleason pattern 4 [5]. Fused glands (FG) are a group of small glands that have lost any intervening stroma [6]. Cribriform glands (CG) are glands composed of a proliferation with multiple punched-out lumina and no intervening stroma, distending a glandular space [6,7]. Figure 1 shows representative examples of G4FG and G4CG morphologies from our cohort. A cribriform pattern is associated with a worse prognosis than poorly formed or fused glands [5] and has increased genomic instability and distinct molecular alterations compared to other patterns as defined by an increased Decipher risk score [8,9]. In the initial 2011 study, the cribriform pattern was present in the majority of cases where prostate-specific antigen (PSA) failure occurred, but only a small number of those where PSA nonfailures occurred (*p* < 0.0001) and had the highest odds ratio for PSA failure compared to other high-grade cancer patterns [10]. Numerous studies since 2011 have shown cribriform glands to be predictive of metastasis and death compared to other high-graded cancers, confirming the aggressiveness of this pattern [4,11,12,13,14].

Multiparametric magnetic resonance imaging (MP-MRI) has shown promise in improving the diagnostic accuracy of high-grade prostate cancer [15,16]. Apparent diffusion coefficient (ADC) images are often used to identify areas of prostate cancer that restrict diffusion [17]. Standardization of the prostate imaging reporting and data system (PI-RADS) has also improved the consistency of clinical radiology reads [18,19]. Recent studies have shown MP-MRI outperforming prostate specific antigen (PSA) testing alone in the identification of clinically significant PCa [20]. Additionally, when MP-MRI is combined with PSA testing and MRI guided biopsy, it outperforms systematic transrectal ultrasound-guided (TRUS) biopsy, therefore reducing unnecessary biopsies that could result in incorrect diagnosis or undertreatment [21]. Noninvasive imaging is becoming more standard for staging and localizing prostate cancer.

Cancer diagnosis involves parallel passing of information between radiology and pathology to downstream physicians. “RadPath” correlates and integrates radiology and pathology reporting to mitigate discordance between these findings [22]. Whole-mount tissue alignment to MP-MRI has enabled non-invasive measurement of pathological features [23,24,25,26]. Recent studies have shown that RadPath techniques can be used to distinguish between Gleason grades, suggesting that MP-MRI features are sensitive to histomorphometric features of prostate cancer [27]. This study sought to determine whether ADC values differed between G4 patterns. We specifically looked at patients with both G4FG and G4CG to identify subject-specific differences in diffusion. In addition, we looked at histomorphometric features to assess gland-level differences between G4 patterns.

## 2. Materials and Methods

### 2.1. Patient Population

Forty-eight patients undergoing MP-MRI prior to prostatectomy were screened for inclusion in this institutional review board (IRB) approved study. Written informed consent was obtained from all patients, ranging in age from 45 to 71 years (mean 61 years). Prior to surgery, a PSA score was measured with an average score of 9.9 ng/mL (range 3.06 to 27.0 ng/mL). Following radical prostatectomy, inclusion criteria required patients to have both G4 cribriform patterned tumors as well as regions of G4 fused gland tumors present within the same slide. This reduced enrollment to 22 patients. Demographic information for all recruited patients and the study cohort is summarized in Table 1.

### 2.2. Imaging and MRI Pre-Processing

MP-MRI was acquired using a 3T MRI scanner (General Electric, Waukesha, WI, USA) using an endorectal coil. Each protocol included T2-weighted imaging, as well as field-of-view (FOV) optimized and constrained undistorted single shot (FOCUS) diffusion weighted imaging (DWI) with ten b-values (0, 10, 25, 50, 80, 100, 200, 500, 1000, and 2000 s/mm^2^).

T2-weighted images were normalized by dividing by the intensity standard deviation within the prostate to correct for inter-subject intensity variation [28]. Apparent diffusion coefficient (ADC) maps were calculated from two combinations of b-values (0–1000 and 1000–2000). The b0 image was aligned with the T2 image using FMRIB’s Linear Image Registration Tool (Functional Magnetic Resonance Imaging of the Brain Library, Oxford, UK). Diffusion maps were then transformed into the T2 space using the calculated transformation matrix [29]. Alignment was verified and manually corrected if misregistration occurred by use of the tkregister tool from FreeSurfer (http://surfer.nmr.mgh.harvard.edu/, accessed on 18 May 2018).

### 2.3. Surgery and Tissue Sectioning

Prostatectomy was performed using the da Vinci robotic system (Intuitive Surgical, Sunnyvale, CA, USA) by a single fellowship-trained surgeon (KMJ) approximately two weeks following imaging [30,31]. Surgical specimens were fixed in formalin overnight, inked, and sectioned using a custom slicing jig [26]. Prostate masks were manually segmented from the patient’s T2-weighted image using AFNI (Analysis of Functional NeuroImages, http://afni.nimh.nih.gov/, accessed on 5 April 2019) [32]. Patient specific slicing jigs were designed using Blender 2.79b (https://www.blender.org/, accessed on 22 March 2018) to match the orientation and slice thickness of each patient’s T2-weighted image [27,33,34,35], and 3D printed using a fifth-generation MakerBot (MakerBot Industries, Brooklyn, NY, USA).

### 2.4. Tissue Segmentation and Annotation

Whole-mount tissue sections were paraffin embedded and slides from the sections were stained for hematoxylin and eosin (H&E). Slides were digitally scanned using a Nikon sliding stage microscope (Nikon Metrology, Brighton, MI, USA). A urological fellowship-trained pathologist (KAI) annotated the whole-mount images using the Gleason grading system [5]. Cribriform glands were distinguished from fused (non-cribriform) glands because of the notable outcome differences between the two types of Gleason 4 tumors [4,10]. Annotations were manually drawn on 4X down-sampled versions of the digitized histology images using a Microsoft Surface Pro 4 (Microsoft, Seattle, WA, USA). Custom code developed in MATLAB (The MathWorks, Natick, MA, USA) automatically segmented lumen and epithelium [34]. An example of an annotated slide and the automated prostate segmentation is shown in Figure 1. This study included a total of 52 whole-mount slides containing both G4FG and G4CG patterns from 22 patients with clinically significant prostate cancer.

### 2.5. Histology Co-Registration

**Figure 2:**
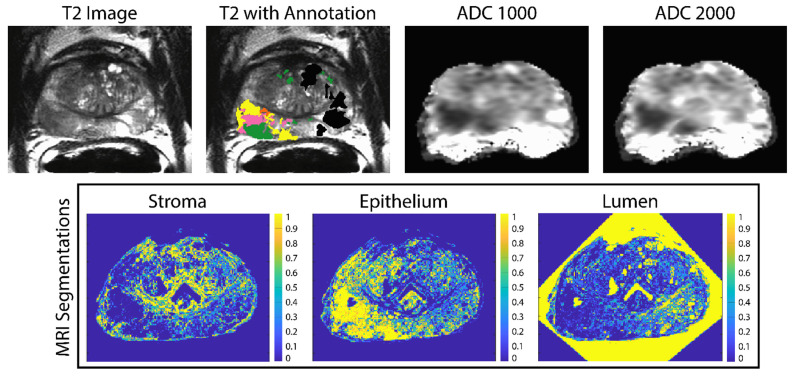
Example of the ADC maps and pathologist annotations aligned to the T2-weighted image (**top**). The annotation key is as follows: G4FG (yellow) G4CG (pink), G3 (green) and benign atrophy (black). The stroma epithelium and lumen segmentations shown on the (**bottom**) are likewise aligned to the T2.

Digitized whole-mount samples were co-registered to the T2-weighted image using previously published software and techniques [25,27,28,33,34,35,36]. A control-point co-registration was applied using manually placed analogous points in each modality, specifically along the boundaries of the organ and on clearly identifiable landmarks within the organ. Approximately 20 to 50 control points were placed on each slide and then down-sampled, along with the whole-mount slide, to MRI resolution. Using MATLAB’s ‘fitgeotrans’ function, a nonlinear spatial transform was calculated from the control points and applied using the ‘imwarp’ function. A local weighted-means transform was used for bringing the histology into MRI space to account for non-uniform distortions caused by compression from the endorectal coil. This transform was additionally applied to the pathologic annotation and segmentations (Figure 2) [33]. A nearest-neighbor interpolation was used for the annotated image to retain the integer values.

### 2.6. Linear Regression Models

To test the hypotheses that ADC and histomorphometric features differed between G4FG and G4CG, a linear mixed model was used. Linear mixed model (LMM) is a method to analyze data that are non-independent and hierarchical which allows both fixed and random effects [37,38,39,40,41]. In this analysis, we fixed a one-layer LMM (1) to consider the different hierarchical structures, considering patient as the random effect. We fixed an additional model (2) considering the nested effect of slide within patient, as can be found in the Supplement. The main effect assessed whether the ADC value and lumen, stromal, and epithelial density differ between G4 patterns. All tests were 2-sided and conducted at the 0.05 significance level. Statistical analyses were performed using R (The R Project for Statistical Computing, https://www.r-project.org, accessed on 18 August 2021) [42]. Specifically, the lme4 package was used for mixed effect models, and the lmerTest package was used for hypothesis testing in the mixed effects models.

## 3. Results

The results from fitted model (1) are shown in Table 2. Both ADC1000 and ADC2000, as well as epithelial density, were observed to be lower in G4CG patterns than G4FG (all *p* < 0.001). Additionally, G4CG presence was associated with higher lumen and stromal densities compared to G4FG (both *p* < 0.001). The random effect estimates the standard deviation for random intercept and error term (residual) for ADC1000 were 0.231 and 0.354, respectively. Results from model (2) (Table S1) demonstrated analogous contrasts with the results from model (1).

## 4. Discussion

In this study, whole-mount tissue slices taken after radical prostatectomy were aligned to the patient’s T2-weighted image to assess both diffusion and histomorphometric differences between Gleason pattern 4 tumors. Linear regression models of ADC values and histomorphometric features found that G4CG was more diffusion-restrictive than G4FG. Additionally, we found that epithelial density was higher in G4CG while lumen and stromal densities were lower compared to G4FG.

Detecting tumor response early in the treatment process is desirable in cancer imaging. Diffusion weighted imaging has been useful for imaging cancer as it shows areas of low water diffusivity non-invasively. Areas of cancer appear darker on ADC images compared to benign tissue. The results of this study suggest that ADC may be lower in G4CG than in G4FG in prostate cancer, suggesting that more deleterious pathologies are associated with a more restricted diffusion within grade groups. These results could help drive surgical intervention, as targeting the darkest areas of the ADC image for biopsy could result in identifying the presence of G4CG and prevent underdiagnosis that could delay patient treatment.

One major limitation of this study is the relatively small patient cohort of 22 patients. Future studies should look in larger populations to provide a more robust understanding of the relationship between diffusion and histomorphometric differences between Gleason pattern 4 tumors. A larger patient cohort could also determine whether machine learning and deep learning applications can further differentiate diffusion in Gleason pattern 4 tumors. Additionally, there were wide ranges in patient Gleason scores and baseline PSA levels. Future studies should determine whether DWI performance varies based on Gleason score and PSA levels at diagnosis, since these analyses were beyond the scope of this study.

We used custom, patient-specific prostate slicing jigs modeled from the T2 image to optimize the orientation of tissue sectioning; however, histologic slides used in this study were cut at 10 μm, averaging 5 slices per patient, sampling a small portion of the full 4-mm MRI slice. While T2-weighted images allow for greater visualization of anatomical landmarks, efforts to align, scale, and resample diffusion maps and annotated regions of G4 tumors to the T2 image may have introduced minor alignment differences between images. Future studies should determine downstream effects on analyses from errors in MRI co-registration.

## 5. Conclusions

We demonstrate in a cohort of 22 patients, that cribriform glands were more diffusion-restricted than fused glands in Gleason pattern 4 tumors within patient. This information may be helpful for clinical decision making both prior to surgery and in radiation treatment dose planning. Future studies should look in larger populations and determine whether other texture features differ between G4 patterns.

## Figures and Tables

**Table 1:** Demographic information for the patient cohort at the time of radical prostatectomy (RP).

	Recruited Patients (*n* = 48)	Study Cohort (*n* = 22)
Age at RP, years (mean, SD)	61 (5.9)	63 (4.3)
Race (*n*, %)		
African American	7 (15)	3 (13)
White/Caucasian	40 (83)	18 (82)
Other	1 (2)	1 (5)
Preoperative PSA, ng/mL (*n*, %)		
≤10	38 (79)	14 (64)
10.1–20.0	8 (17)	6 (27)
≥20	2 (40	2 (9)
Grade group at RP (*n*, %)		
6	9 (19)	1 (5)
3 + 4	22 (46)	11 (50)
4 + 3	6 (12)	4 (18)
8	8 (17)	4 (18)
≥9	3 (6)	2 (9)
pT (*n*, %)		
1	33 (69)	14 (64)
2	11 (23)	6 (27)
3	4 (8)	2 (9)
Gleason 4 Subtypes (*n*, %)		
Cribriform glands	27 (56)	22 (100)
Fused glands	40 (83)	22 (100)

**Table 2:** One-Layer LMM considering patient as random effect. Estimate and 95% Confidence Intervals given in units of mm^2^/s.

ADC1000	**Fixed effect**	**Estimate**	**95% CI**	**t-value**	**Pr (>|t|)**
	Intercept	1.274	(1.176, 1.373)	25.88	<0.0001
	Cribriform vs. Fused Glands	−0.096	(−0.103, −0.089)	−26.682	<0.0001
	**Random effect**	**Std Dev**	**95% CI**		
	Subject (Intercept)	0.231	(0.172, 0.313)		
	Residual	0.354	(0.352, 0.355)		
ACD2000	**Fixed effect**	**Estimate**	**95% CI**	**t-value**	**Pr (>|t|)**
	Intercept	0.933	(0.862, 1.003)	26.601	<0.0001
	Cribriform vs. Fused Glands	−0.062	(−0.066, −0.057)	−26.428	<0.0001
	**Random effect**	**Std Dev**	**95% CI**		
	Subject (Intercept)	0.164	(0.123, 0.223)		
	Residual	0.229	(0.228, 0.230)		
Lumen	**Fixed effect**	**Estimate**	**95% CI**	**t-value**	**Pr (>|t|)**
	Intercept	0.0515	(0.041, 0.062)	9.755	<0.0001
	Cribriform vs. Fused Glands	0.0173	(0.015, 0.02)	12.295	<0.0001
	**Random effect**	**Std Dev**	**95% CI**		
	Subject (Intercept)	0.0244	(0.018, 0.033)		
	Residual	0.138	(0.138, 0.139)		
Stroma	**Fixed effect**	**Estimate**	**95% CI**	**t-value**	**Pr (>|t|)**
	Intercept	0.831	(0.799, 0.863)	51.682	<0.0001
	Cribriform vs. Fused Glands	0.094	(0.088, 0.099)	35.276	<0.0001
	**Random effect**	**Std Dev**	**95% CI**		
	Subject (Intercept)	0.0749	(0.056, 0.102)		
	Residual	0.260	(0.259, 0.261)		
Epithelium	**Fixed effect**	**Estimate**	**95% CI**	**t-value**	**Pr (>|t|)**
	Intercept	0.118	(0.088, 0.147)	7.971	<0.0001
	Cribriform vs. Fused Glands	−0.111	(−0.115, −0.106)	−50.11	<0.0001
	**Random effect**	**Std Dev**	**95% CI**		
	Subject (Intercept)	0.0689	(0.051, 0.094)		
	Residual	0.217	(0.216, 0.218)		

## Data Availability

The data presented in this study are available on request from the corresponding author. The data are not publicly available due to privacy concerns.

## Supplementary Materials

The following supporting information can be downloaded at: https://www.mdpi.com/article/10.3390/tomography8020053/s1, Table S1: Two-Layer LMM considering the slides nested within patients as the random effect. Estimate and 95% Confidence Intervals given in units of mm^2^/s.
