# Supplementary material for: Diffusion Restriction Comparison between Gleason 4 Fused Glands and Cribriform Glands within Patient Using Whole-Mount Prostate Pathology as Ground Truth
Source: Tomography. 2022 Mar 2;8(2):635–43. doi: 10.3390/tomography8020053 (PMC8938782; doi:10.3390/tomography8020053)
Supplement: Supplementary file 1 [file tomography-08-00053-s001.zip › tomography-1577541-supplementary.pdf]

Supplement

Table S1. Two-Layer LMM considering the slides nested within patients as the random effect. Estimate and 95% Confidence Intervals given in units of mm^2/s.

<table>
<tr><td rowspan="7">ADC1000</td><td>Fixed effect</td><td>Estimate</td><td>95% CI</td><td>t-value</td><td>Pr(>|t|)</td></tr>
<tr><td>Intercept</td><td>1.311</td><td>(1.203, 1.419)</td><td>24.288</td><td><0.0001</td></tr>
<tr><td>Cribriform vs Fused Glands</td><td>-0.062</td><td>(-0.069, -0.055)</td><td>-18.013</td><td><0.0001</td></tr>
<tr><td>Random effect</td><td>Std Dev</td><td colspan="2">95% CI</td><td rowspan="4"></td></tr>
<tr><td>Subject:Slide (Intercept)</td><td>0.247</td><td colspan="2">(0.213, 0.290)</td></tr>
<tr><td>Subject (Intercept)</td><td>0.222</td><td colspan="2">(0.144, 0.321)</td></tr>
<tr><td>Residual</td><td>0.287</td><td colspan="2">(0.286, 0.288)</td></tr>
<tr><td rowspan="7">ACD2000</td><td>Fixed effect</td><td>Estimate</td><td>95% CI</td><td>t-value</td><td>Pr(>|t|)</td></tr>
<tr><td>Intercept</td><td>0.959</td><td>(0.878, 1.041)</td><td>23.58</td><td><0.0001</td></tr>
<tr><td>Cribriform vs Fused Glands</td><td>-0.033</td><td>(-0.038, -0.029)</td><td>-14.621</td><td><0.0001</td></tr>
<tr><td>Random effect</td><td>Std Dev</td><td colspan="2">95% CI</td><td rowspan="4"></td></tr>
<tr><td>Subject:Slide (Intercept)</td><td>0.170</td><td colspan="2">(0.147, 0.200)</td></tr>
<tr><td>Subject (Intercept)</td><td>0.172</td><td colspan="2">(0.115, 0.245)</td></tr>
<tr><td>Residual</td><td>0.189</td><td colspan="2">(0.188, 0.190)</td></tr>
<tr><td rowspan="7">Lumen</td><td>Fixed effect</td><td>Estimate</td><td>95% CI</td><td>t-value</td><td>Pr(>|t|)</td></tr>
<tr><td>Intercept</td><td>0.0471</td><td>(0.039, 0.056)</td><td>10.851</td><td><0.0001</td></tr>
<tr><td>Cribriform vs Fused Glands</td><td>0.0247</td><td>(0.022, 0.028)</td><td>15.456</td><td><0.0001</td></tr>
<tr><td>Random effect</td><td>Std Dev</td><td colspan="2">95% CI</td><td rowspan="4"></td></tr>
<tr><td>Subject:Slide (Intercept)</td><td>0.0426</td><td colspan="2">(0.0370, 0.0490)</td></tr>
<tr><td>Subject (Intercept)</td><td>0</td><td colspan="2">(0, 0.0170)</td></tr>
<tr><td>Residual</td><td>0.135</td><td colspan="2">(0.134, 0.135)</td></tr>
<tr><td rowspan="7">Stroma</td><td>Fixed effect</td><td>Estimate</td><td>95% CI</td><td>t-value</td><td>Pr(>|t|)</td></tr>
<tr><td>Intercept</td><td>0.815</td><td>(0.777, 0.851)</td><td>45.071</td><td><0.0001</td></tr>
<tr><td>Cribriform vs Fused Glands</td><td>0.0697</td><td>(0.064, 0.075)</td><td>24.470</td><td><0.0001</td></tr>
<tr><td>Random effect</td><td>Std Dev</td><td colspan="2">95% CI</td><td rowspan="4"></td></tr>
<tr><td>Subject:Slide (Intercept)</td><td>0.170</td><td colspan="2">(0.146, 0.198)</td></tr>
<tr><td>Subject (Intercept)</td><td>0.0304</td><td colspan="2">(0, 0.090)</td></tr>
<tr><td>Residual</td><td>0.238</td><td colspan="2">(0.237, 0.239)</td></tr>
<tr><td rowspan="7">Epithelium</td><td>Fixed effect</td><td>Estimate</td><td>95% CI</td><td>t-value</td><td>Pr(>|t|)</td></tr>
<tr><td>Intercept</td><td>0.140</td><td>(0.103, 0.179)</td><td>7.471</td><td><0.0001</td></tr>
<tr><td>Cribriform vs Fused Glands</td><td>-0.0941</td><td>(-0.0990, -0.09)</td><td>-40.486</td><td></td></tr>
<tr><td>Random effect</td><td>Std Dev</td><td colspan="2">95% CI</td><td rowspan="4"></td></tr>
<tr><td>Subject:Slide (Intercept)</td><td>0.163</td><td colspan="2">(0.140, 0.192)</td></tr>
<tr><td>Subject (Intercept)</td><td>0.0429</td><td colspan="2">(0, 0.0980)</td></tr>
<tr><td>Residual</td><td>0.194</td><td colspan="2">(0.193, 0.195)</td></tr>
</table>
